# Adaptive immunology of *Cryptococcus neoformans* infections—an update

**DOI:** 10.3389/fimmu.2023.1174967

**Published:** 2023-05-11

**Authors:** Junsong Chen, Jiasheng Shao, Min Dai, Wei Fang, Ya-li Yang

**Affiliations:** ^1^ Key Laboratory of Systems Biomedicine (Ministry of Education), Shanghai Center for Systems Biomedicine, Shanghai Jiao Tong University, Shanghai, China; ^2^ Department of Immunology and Rheumatology, Jiading District Central Hospital Affiliated Shanghai University of Medicine & Health Science, Shanghai, China; ^3^ Tulane National Primate Research Center, Tulane University School of Medicine, Covington, LA, United States; ^4^ Department of Laser and Aesthetic Medicine, Shanghai Ninth People’s Hospital, Shanghai JiaoTong University School of Medicine, Shanghai, China; ^5^ Department of Dermatology, Shanghai Ninth People’s Hospital, Shanghai JiaoTong University School of Medicine, Shanghai, China

**Keywords:** *Cryptococcus neoformans*, cryptococcosis, adaptive immunity, B cells, T cells, cytokines

## Abstract

The fungal genus *Cryptococcus* comprises a group of pathogens with considerable phenotypic and genotypic diversity that can lead to cryptococcosis in both healthy and immunocompromised individuals. With the emergence of the HIV pandemic, cryptococcosis, mainly meningoencephalitis, afflicts HIV-infected patients with severe dysfunction of T cells. It has also been reported in recipients of solid organ transplantation and in patients with autoimmune diseases who take immunosuppressive agents long-term, as well as in those with unidentified immunodeficiency. The clinical outcome of the disease is primarily determined by the immune response resulting from the interplay between the host immune system and the pathogen. Most human infections are caused by *Cryptococcus neoformans*, and nearly all immunological studies have focused on *C. neoformans*. This review provides an updated understanding of the role of adaptive immunity during infection with *C. neoformans* in human and animal models over the past half-decade.

## 1 Introduction

Cryptococcal meningitis (CM) is a common fungal infectious disease of the central nervous system (CNS) caused by *Cryptococcus neoformans* and is a severe fungal disease that endangers human health. It is responsible for approximately 200,000 global deaths annually (1). *C. neoformans*, also known as *Torula histolytica*, is an encapsulated yeast that can survive in soil, pigeons, milk, and fruits. It can infect humans and animals and typically manifests as an exogenous infection; however, it may also be endogenous and opportunistic.

Cryptococcal disease, or cryptococcosis, is caused by the *Cryptococcus* species complex and can occur in both immunocompromised patients and healthy individuals. The *Cryptococcus* complex comprises seven species (2). *C. neoformans* and *Cryptococcus deneoformans* primarily cause infections in severely immunocompromised individuals, including patients with HIV/AIDS, especially those with CD4^+^ T-cell counts of <50 cells/μl (3, 4). More than 90% of patients with CM have been identified as patients with HIV/AIDS, especially those with CD4^+^ T-cell counts of <100 cells/ml, those with various T-cell deficiencies associated with disorders such as chronic respiratory, liver, and kidney diseases; and patients receiving immunosuppressive treatments before organ transplantation (5, 6). Five species of *Cryptococcus gattii* may cause disease in immunosuppressed and immunocompetent individuals (5). Approximately 60% of the affected population has underlying medical conditions, such as respiratory disease, diabetes, and hematological malignancies, during infection with *C. gattii* (7). These discrepancies indicate that *Cryptococcus* species may exhibit nuances in their interactions with their hosts. The vast majority of studies exploring immune responses have been done on *C. neoformans* infections in human and animal models.

Both innate and adaptive immune systems play important roles in the host’s defense against *C. neoformans*. The first line of defense against microbes is the surface barrier(s) of the innate immune system (e.g., nasal mucosa and skin). In addition, human serum and saliva exhibit anticryptococcal activity. More importantly, acquired immune mechanisms contribute significantly to resistance to cryptococcosis (2, 8). This review will mainly focus on the advances in host adaptive immunity [CD4^+^ T cells, CD8^+^ T cells, gamma delta (γδ) T cells, regulatory T cells (Tregs), B cells, and T-cell-derived cytokines] against *C. neoformans* infection in recent years.

## 2 CD4^+^ T cells

CD4^+^ T cells are important components of host-mediated cellular immunity. They can be classified into the T helper 1 (Th1), Th2, and Th17 subgroups according to the characteristics of the cytokines and effector cells produced. The virulence factors of *C. neoformans* can destroy the host immune system and cause disease, and their mechanism of action is closely related to the Th2-type immune response. The major virulence factors of *C. neoformans*—urease, laccase, and capsules—promote the aggregation of immature dendritic cells (DCs) and induce a non-protective Th2 immune reaction (9, 10). In mice, CD4^+^ T cells play a dominant role in cell-mediated immunity against *C. neoformans*. Through adaptive immunity, CD4^+^ T cells promote the transcription and expression of the related cytokines and chemokines, orchestrate fungal clearance, and confer protection to naive mice (8, 11). A CD4^+^ T-cell count lower than 100 cells/μl and detectable cryptococcal polysaccharide capsule antigen (CrAg) in the serum indicate an increased risk of HIV-related *C. neoformans* infection (12). Monitoring the CD4^+^ T-cell count in peripheral blood is a convenient and reliable method to predict the severity and clinical outcomes of patients with cryptococcosis. Cryptococcus can also appear in association with idiopathic CD4^+^ T-cell cytopenia without HIV infection, and the chance of disseminated cryptococcosis is high in patients with a CD4^+^ T-cell count lower than 400 cells/μl (13). Recent studies have suggested that routine screening of HIV-infected patients with a CD4^+^ T-cell count <100 cells/μl for cryptococcal infection can prevent CM in both resource-limited and high-income countries (14).

Although CD4^+^ T cells play a role against cryptococcal infection, they could also augment the immunopathological reaction and the Th1-biased response with prominent upregulation of pro-inflammatory cytokines. This results in high mortality due to infection of the CNS with *C. neoformans* in both HIV-positive and HIV-negative patients (15). CNS infections include the development of immune reconstitution inflammatory syndrome (IRIS) in immunocompromised patients and post-infectious inflammatory response syndrome (PIIRS) in immunocompetent patients. Several studies have reported that high serum interleukin 4 (IL-4) levels are associated with the onset of HIV infection, IRIS/PIIRS, and death. This finding may support the development and use of anti-inflammatory therapies to minimize CNS damage in patients with severe cryptococcosis (16). In patients with advanced symptomatic HIV infection (CD4^+^ T-cell count <100 cells/μl), fluconazole therapy has not been shown to particularly increase the survival rate when compared to newly diagnosed individuals (17).

## 3 CD8^+^ T cells

In addition to CD4^+^ T cells, CD8^+^ T cells play a predominant role in host immunity against *C. neoformans* infection by producing tumor necrosis factor gamma (TNF-γ) to inhibit and kill the pathogen *via* direct contact. The absence of CD4^+^ T cells will lead to the proliferation of CD8^+^ T cells, and CD8^+^ T cells compensate and defend CD4^+^-depleted hosts from an otherwise fatal infection (18).

Interleukin 17A (IL-17A), which is secreted by CD8^+^ T cells, mediates the immune response and protects the host from fatal fungal infections (19). CD8^+^ T cells also have the capability to mediate the direct killing of *C. neoformans*, by which the TNF-γ produced by CD8^+^ T cells can curb the growth and survival of *C. neoformans* in macrophages.

CD8^+^ T cells also produce TNF-α and Th1 cytokines during cryptococcal infections, whereas CD4^+^ T cells secrete only Th2 cytokines. Peripheral blood mononuclear cells (PBMCs) in men have lower adaptive immunity-related (CD3^+^, CD4^+^, and CD8^+^) T-cell percentages during cryptococcal infection than in women. This may explain the disparity in the incidence of cryptococcosis between men and women (20). Antiretroviral therapy (ART) was found to alter the clonotypic phenotype and the structural composition of adaptive immunity in HIV-infected patients with CM. After ART, the CD8^+^ central memory cell subset gradually declined compared to the CD4^+^ T-cell population. Both the immune exhaustion of CD8^+^ T cells and the activation markers remained elevated over 12 weeks (21).

## 4 Gamma delta T cells

In a lot of organs containing epithelia, γδ T cells modify the inflammatory responses and might play an important role in antifungal host immune defense by acting as the first line of defense at the mucosal level. For instance, *C. neoformans* infection in the lung induces the increase of γδ T cells and eliminates the fungal pathogen (22). γδ T cells gather in the lungs during pulmonary *C. neoformans* infection, which does not require the involvement of the MCP-1 chemokine. After cryptococcal infection, γδ T cells secrete IL-17A and interferon gamma (IFN-γ) in mature neutrophils. T cells produce IL-17 without antigen induction (23). IL-17A is primarily produced by neutrophils. However, neutrophils are negatively regulated by γδ T cells that produce IL-17A. When neutrophils are absent, γδ T cells can produce IL-17A cytokines, which are related to the protective immune response. Administration of the *C. neoformans* Δ*sgl1* mutant vaccine can accumulate sterylglucosides (SGs) and produce normal capsules, which protect mice from *C. neoformans* infection even when CD4^+^ T cells are deficient (24). γδ T cells also downregulate the Th1 response and develop host resistance against *C. neoformans* infection (25).

## 5 Regulatory T cells

Tregs are a subset of CD4^+^ T cells that are critical mediators of peripheral tolerance and are immune response modulators that usually inhibit autoimmune reactions. Their development depends on the expression of the transcription factor *FoxP3*, and the maintenance of their suppressive function specifically requires the expression of IL-33 (26–28).

Tregs play both positive and negative roles in fungal infections. They disrupt the dynamic balance of Th1/Th2/Th17 cells, reduce the expression of Th2 cytokines, and inhibit the differentiation of Th17 cells. In Treg-deficient mice, the production of immunoglobulin E (IgE), eosinophils, and Th2 cytokines (IL-4, IL-5, and IL-13) is increased. Furthermore, the fungal load and mucus production in mice increases significantly with increased pulmonary allergic inflammation. Although Th1 cells play a protective role, Th2 cells can aggravate cryptococcal diseases. However, the mechanism underlying the Treg-mediated inhibition of Th2 cells remains unclear. The secretion of IL-10 by Tregs is a well-known method for inhibiting the response of lung Th2 cells. However, in a mouse model of cryptococcal infection, inhibition of Th2 cells by blocking IL-10 had little effect on infection control. A murine model of experimental cryptococcosis showed the accumulation of Tregs in the lung parenchyma rather than in the draining lymph nodes during infection. Tregs inhibit the Th2 immune response by co-localizing C–C chemokine receptor type 5 (CCR5) and IFN regulatory factor 4 (IRF4) with Th2 effector cells in the lung tissue, thereby boosting fungal control (29). The main function of Tregs during cryptococcal infection is to inhibit Th2 cells. Therefore, Tregs are promising targets for the immunomodulatory treatment of infections.

Allergic airway inflammation caused by fungal sensitization is a challenging problem because it is difficult to treat and it leads to serious illnesses. A reduction in the number of Tregs results in stronger allergic reactions, with increased Th2 cytokine responses and more active fungal proliferation. Application of the IL-2/anti-IL-2 complex during *C. neoformans* infection causes a transient increase in Tregs *in vivo*, which can reduce the level of IgE, the secretion of Th2 cytokines in the serum, and the occurrence of allergic airway inflammation. Therefore, a combination of the IL-2/anti-IL-2 complex and antifungal agents could be used in the treatment of allergic airway disorders in the future (30). Tregs may be potential therapeutic targets for the prevention or reduction of fungus-related airway diseases.

## 6 B cells

HIV infection is a vital risk predictor for CM, emphasizing the significant role of T-cell-mediated immunity in disease prevention. However, over the past decade, numerous studies have demonstrated that antibody immunity plays a role in resistance to CM. In mice, B cells reduce the early spread from the lungs to the brain, and IgM in naive mice can strengthen fungal infections in the lungs. A previous study also confirmed the potential role of B cells and certain antibodies in the natural resistance to CM (31).

A retrospective study on banked peripheral blood lymphocytes from HIV-positive samples indicated that those who suffered from cryptococcosis had lower levels of IgM memory (CD19^+^CD27^+^IgM^+^) B cells than those who did not. Therefore, B-cell deficiency may be a relevant factor in *C. neoformans* infection. An animal experiment demonstrated that the adoptive transfer of B cells from naive wild-type mice to Rag1^−/−^ mice halted the early dissemination of *C. neoformans* to the brain. These data also demonstrate that naive B cells could intervene with a decrease in fungal dissemination to the brain in Rag1^−/−^ mice (32). One study examined the levels of *C. neoformans*-specific antibodies in the serum and peripheral blood B-cell subsets in 12 previously healthy patients with cryptococcosis and 21 controls. After adjusting for race, sex, and age, the cryptococcal capsular polysaccharide IgG levels were found to be higher in patients than in controls, but the levels of total B cells and memory B cells were lower. These results confirm previous findings in patients with HIV-associated cryptococcosis and indicate that perturbations in the B-cell subsets may also be associated with the disease in previously normal patients with cryptococcosis. Their experiment also showed that the levels of total memory and IgM memory B cells decreased in previously healthy patients with cryptococcosis compared to controls. These findings support and validate previous reports on cryptococcosis under conditions of B-cell deficiency and HIV infection and on animal data linking B-1 cells to resistance to *Cryptococcus* spp (33).

## 7 Cytokines and correlation with immunity

Cytokines are a group of small molecules that interact and communicate with different types of immune cells. They play a crucial role in protecting mice from cryptococcal infections. A common characteristic of these protective cytokines and chemokines is that they induce the immune response of Th1, enhance the activity of other Th1-inducing cytokines, and suppress the immune response of Th2 (2).

The Th1-type responses produce IL-2, IL-12, IFN-γ, and TNF-γ. IL-12, IFN-γ, and TNF-α can protect against cryptococcosis, whereas a reduction in IL-2, IL-12, and IFN-γ can increase the risk of pulmonary and cerebral fungal infection. Early induction of TNF-α contributes to the classical activation pathway of DCs, followed by the generation of a protective immune response during the dissemination phase of *C. neoformans* infection through the accumulation of T cells and an improved balance of Th1/Th2 cytokines. TNF-α is a mononuclear factor released by mononuclear macrophages in the early stage of *C. neoformans* infection. TNF-α has been demonstrated to promote the proliferation and differentiation of lymphoid cells, decrease the activation of M2-type macrophages, and reduce pulmonary eosinophilia and alternative activation of lung macrophages during the adaptive phase of infection (34). Patients living with HIV/AIDS exhibit enhanced activation of NF-κB and TNF-α mRNA transcription following cryptococcal infection, which can regulate the expression of TNF-α. The risk of *C. neoformans* infection is markedly increased in patients with sepsis, cancer, and autoimmune diseases (35). In patients with HIV/AIDS and cryptococcal meningoencephalitis, the levels of IL-2, IL-12, and IFN-γ could decrease the fungal load in the cerebrospinal fluid (CSF) and therefore improve patients’ clinical outcomes.

*C. neoformans* also induces the production of other cytokines, such as IL-6, IL-1α, IL-1β, and type I interferon (IFN-I) from innate immune cells, of which IL-6 can induce the activation of STAT3 phosphorylation, which plays a crucial role in acute/chronic inflammation and fungal infection (36). IL-1α and IL-1β are pro-inflammatory cytokines that are secreted after *C. neoformans* infection and activate the interleukin-1 receptor type I (IL-1RI), whereas the IL-1RI signaling pathway is indispensable for the activation of both the innate and adaptive immunities that enhance host defense and survival after *C. neoformans* infection in mice (37, 38). IL-17 is a pro-inflammatory cytokine that plays an important role in antifungal immunity. For instance, the IL-17 pathway initiates and regulates fungal immunity by upregulating the pro-inflammatory cytokines, antimicrobial peptides, and neutrophil chemokines. Compared to patients with tuberculous meningitis or non-neurosyphilis, patients with AIDS- or non-AIDS-associated CM had significantly higher levels of IL-17 in the CSF. IL-17A is produced during the innate immune response and the early stages of *C. neoformans* infection. IL-17A does not exert its effect but instead leads to a reduced Th1 immune response and attenuates the host defense against *C. neoformans* infection. In the later phases of cryptococcal infection, leukocytes are recruited and activated, and IFN-γ is produced to enable the containment and clearance of *C. neoformans*. IL-17 is mainly produced by CD4^+^ T cells stimulated by *C. neoformans*, and its production is dependent on JAK2/STAT3 signaling. *C. neoformans*-mediated IL-17 expression is attenuated by the inhibition of STAT3 phosphorylation. In human studies, the enhancement of IL-17 results in enhanced fungal clearance and improved clinical outcomes (39).

IL-23 is an important molecule upstream of IL-17 that promotes Th17 cell activation proliferation and inflammatory cytokine secretion. IL-23 suppresses allergic responses to cryptococcal infections through the IL-17-independent inhibition of eosinophil recruitment and the IL-17-dependent regulation of antibody production and eosinophilic crystal deposition (40). In animal models, IL-23 exerts a protective effect against cryptococcal infection, and the loss of IL-23 leads to impaired chemotactic and cytokine responses in inflammatory cells, providing a potential therapeutic target for immunization against *C. neoformans*.

In general, Th2 cytokines including IL-4, IL-5, and IL-13 inhibit immune protection and exacerbate cryptococcal diseases. In animal models, high levels of IL-4 slow the clearance of intracellular *C. neoformans* and increase the mortality of infected mice. Both IL-5 and IL-13 promote the aggregation and activation of pulmonary eosinophils, thereby increasing the fungal burden and overall sensitivity to *C. neoformans* infection (41, 42). IL-25, a type 2 cytokine produced by epithelial cells, contributes to the pathogenesis of cryptococcosis. Infection with the highly virulent *C. neoformans* strain can induce significant expression of IL-25 in the lungs, but not in the brain. IL-25 signal transduction inhibits the expression of the cytokines and chemokines related to the protection of the brain, including *Ifng*, *Il1b*, *Ip10*, and *Nos2*, but does not directly affect brain cell inflammation or microglial activation. IL-25 signal transduction inhibits protective immunity and improves the type 2 immune response, which may be beneficial in the development of cryptococcal diseases in the CNS (43). IL-33 is widely expressed in different tissues and cells of the human body, including inflammatory cells such as mast cells, macrophages, and DCs, as well as non-inflammatory cells such as endothelial cells, epithelial cells, and fibroblasts, *via* a unique receptor composed of T1/ST2 (homolog of sulfotransferase 2) and the IL-1R accessory protein. *C. neoformans* infection increased the expression of IL-33 in lung tissues, with the accumulation of type 2 pulmonary innate lymphoid cells. Compared to wild-type mice, IL-33R subunit T1/ST2-deficient mice infected with *C. neoformans* showed decreased fungal burden in the lungs, spleen, and brain and improved survival. Wang et al. also found that, in the early stages of *C. neoformans* infection, the load of pulmonary fungi in IL-33^−/−^ and ST2^−/−^ mice decreased with an increase in neutrophil infiltration (44). These results indicate that IL-33-dependent signal transduction contributes to the expansion of congenital type 2 immunity and the subsequent Th2-biased lung immunopathology, thereby promoting the growth and transmission of *C. neoformans* (45). Th17 cells are involved in both the protection from and the enhancement of disease during *C. neoformans* infection. Galactoxylose mannan (GalXM) is an effective DC activator. When co-cultured with T-cells, it induces a Th17 cytokine response. Treatment of mice with GalXM before infection with *C. neoformans* protected them from infection, and this effect was dependent on the levels of IL-6 and IL-17 (46).

As a potent anti-inflammatory and immunosuppressive cytokine, IL-10 is pivotal for maintaining peripheral immunological tolerance and inducing inflammatory tissue damage (47). DCs coordinate critical transitions from innate to adaptive immunity, and the blockade of IL-10 signaling improves fungal clearance in mouse models of cryptococcal lung infection (48). IL-17 may serve as a diagnostic biomarker for *C. neoformans* infection, while STAT3 holds promise as a potential checkpoint for targeted antifungal therapy. IL-12 treatment in mice infected with *C. neoformans* prevents systemic cryptococcosis, particularly cryptococcal meningoencephalitis. In addition to conventional antifungal therapies, these treatment options may become important adjunct therapies in the future.

## 8 Progress in clinical research

Current clinical treatment of cryptococcosis relies on the following three classes of antifungal agents: polyenes, such as amphotericin B and nystatin, triazoles, such as voriconazole and itraconazole, and nucleoside drugs, such as 5-fluorocytosine (49). Triazoles have the advantages of a broad spectrum, high efficacy, few toxic effects, and easy passage through the blood–brain barrier. Moreover, they can reach high concentrations in the CSF and are therefore often used to treat CM. However, in the presence of azoles, *C. neoformans* tends to undergo point mutations, generating resistant strains in clinical settings. In addition, 5-fluorocytosine alone causes drug resistance, severe hepatotoxicity, and myelosuppression. These issues make the clinical use of these drugs challenging (50). In response to the increasingly severe form of *C. neoformans* infection in clinical settings, new therapeutic research directions have been focused on the following: gene-targeted therapy and the development of targeted therapeutic approaches at the molecular level targeting the key sites involved in the synthesis of virulence factors such as the capsule, Titan cells, and melanin involved in the process of *C. neoformans* infection (51).

CRISPR/cas9 gene therapy is used to inhibit or block the synthesis of virulence factors to treat *C. neoformans* infections (52). Immunotherapy, which harnesses immunomodulatory factors, is an important developmental strategy for *C. neoformans* treatment. The combination of antibodies with radioactive molecules enhances the efficacy of antifungal antibodies *via* radioimmunotherapy. IFN-γ has been utilized to induce the repolarization of M2-type macrophages into M1-type macrophages to elevate antifungal activity (53). CD8^+^ T cells can compensate for the lack of CD4^+^ T cells, suggesting that adaptive immune cells may serve as immunotherapeutic targets. During vaccine development, the artificial glucuronoxylomannan (GXM) epitope mimetic peptide P13 mimicked the GXM polysaccharide configuration of the capsule, thereby inducing an effective protective immune response in the host (54). GXM monoclonal antibodies (mAbs) have been shown to counteract *C. neoformans* invasion in classical ways, such as by enhancing phagocytosis, activating the complement system, recruiting inflammatory cells, and acting directly on GXM. The 18b7 mAb protects the host by altering the capsular architecture and physical properties (55). β-dextrans or the immunodominant proteins extracted from *C. neoformans* may be the relevant antigenic stimuli (56). Mice administered with the mannoprotein vaccine showed increased survival after *C. neoformans* infection (57). Small-molecule drug-targeted therapies, such as haloquinoline molecular analogs, can counteract *C. neoformans* and affect biofilm maturation. Furthermore, they can bind to exogenous ergosterol and cholesterol, counteracting their biological behavior by influencing the cell membrane stability of *C. neoformans* (58).

## 9 Conclusion

*C. neoformans* can be easily found in the environment globally, and humans are commonly exposed to it. However, diseases with apparent clinical symptoms are rare, except in immunocompromised patients. The prevalence of HIV/AIDS predominantly enhances the occurrence of disseminated cryptococcosis, which also occurs in individuals with other types of immune impairments attributable to immunosuppressive therapy administered before solid organ transplantation and to autoimmune diseases (59, 60). However, some patients with cryptococcal disease do not have identified predisposing conditions. Interpretation of the adaptive immune response to *C. neoformans* primarily originates from studies using murine models. The accessibility of transgenic mouse strains makes them vital tools for exploring different facets of the interactions between the host immune system and pathogens. However, the findings in mice are not always similar to those in humans. For instance, the Th2 immune response is related to an increased disease burden of *C. neoformans* in mice (61); however, some studies in humans have not linked the Th2 immune response to disease enhancement (62, 63). One explanation for this difference could be the different types of specimens: CSF and blood from humans and lung tissues from mice. In addition, we could not determine the protective immunity in healthy individuals because human studies have been restricted to patients with *C. neoformans* infections. At present, there is no recognized and effective immunotherapy program in the field of cryptococcosis treatment; however, some studies have shown that cytokines can be used for the targeted treatment of cryptococcal infections, including the development of immunotherapy-targeted cells and antifungal vaccines.

In many countries, especially in those with high HIV seroprevalence, CM, the major clinical manifestation of cryptococcal disease, is associated with high morbidity and mortality (64), which has led to studies in humans. Although the lungs are the first site of cryptococcal invasion, the recognition and immune responses of human lungs to *C. neoformans* still need to be elucidated. Studies in mice have shown that alterations in the morphology or mitochondria of *C. neoformans* can affect its virulence and stress resistance (2, 65). In particular, the increase in the chitin content in the cell wall is related to the harmful Th2 immune response in the lungs, which aggravates cryptococcosis, whereas Tregs aggregate in the lungs independent of priming in the draining lymph nodes.

It is now known that alterations in cell surface components affect human immunity against *C. neoformans* (20). Several studies have been conducted to elucidate the origin and role of the different types of cytokines in anti-cryptococcal immune responses in murine models (2, 34, 41). However, the functions of these cytokines in infections in humans are not well understood.

Different cytokines have been associated with either the improvement of patient survival or the worsening of the disease; however, their specific roles in immunity against *C. neoformans* are not fully understood. Numerous different adaptive immune components and functions, including T cells, B cells, and cytokines, contribute to the host defense against *C. neoformans*; however, the absence of a single constituent (e.g., CD4^+^ T cells) appears insufficient to cause human cryptococcal disease (18). New insights into the susceptibility and resistance to cryptococcosis may provide a framework for the development of new drugs for the prevention and treatment of this disease. However, a major challenge is ensuring the global accessibility of cART (chimeric antigen receptor T) and antifungal drugs, especially in those areas where HIV/AIDS is still prevalent. The administration of fluconazole and the initiation of cART can reduce the burden of HIV-related cryptococcosis. Prompt diagnosis and easy access to these drugs are necessary to overcome cryptococcosis in resource-limited settings.

The clinical outcome is based on the immune status of the patient, which interacts with *C. neoformans* and the host, indicating that methods that enhance host immunity are logical, especially in patients with known defects. Immunotherapy, including vaccines and mediators, such as TNF-α or IFN-γ, antibody-based treatment, and prophylaxis, are promising modalities for the prevention and treatment of cryptococcosis. It is optimal to develop a vaccine that can prevent the acute onset of the disease, as well as reactivation, owing to the ubiquitous nature of *C. neoformans* in the environment. Excessive or insufficient immune responses may lead to cryptococcal disease, and more than one type of vaccine may be required. Over the past two decades, a large amount of information on host immunity against *C. neoformans* has been obtained, which has accelerated vaccine development. In this respect, some vaccine candidates and platforms have been developed. To date, the most promising vaccines are GXM protein conjugates and whole-cell vaccines that promote the production of IFN-γ. Vaccines that boost innate immunity and leverage the immunogenicity of the cell wall components are still under development. Although significant progress has been made in understanding the host immune response to *C. neoformans* over the past three decades, the challenge is to exploit this knowledge in order to identify new biomarkers of infection risk and to prevent and treat cryptococcosis using novel therapeutic modalities or vaccines that bolster host immunity against the pathogen.

## Data availability statement

The original contributions presented in the study are included in the article/supplementary material. Further inquiries can be directed to the corresponding authors.

## Author contributions

All authors listed have made a substantial, direct, and intellectual contribution to the work, and approved it for publication.
